# Modulation of xylanase production from alkaliphilic *Bacillus pumilus* VLK-1 through process optimization and temperature shift operation

**DOI:** 10.1007/s13205-013-0160-2

**Published:** 2013-08-18

**Authors:** Lalit Kumar, Davender Kumar, Sushil Nagar, Rishi Gupta, Neelam Garg, Ramesh Chander Kuhad, Vijay Kumar Gupta

**Affiliations:** 1 Department of Biochemistry, Kurukshetra University, Kurukshetra, 136119 Haryana India; 2 Department of Microbiology, Kurukshetra University, Kurukshetra, 136119 Haryana India; 3 Lignocellulose Biotechnology Laboratory, Department of Microbiology, University of Delhi South Campus, Benito Juarez Road, New Delhi, 110021 India

**Keywords:** Wheat straw, Temperature shift, Saccharification, Response surface methodology, Xylanase

## Abstract

This study was aimed at enhancing the production of xylanase from an alkaliphilic *Bacillus pumilus* VLK-1 in submerged fermentation using wheat bran, a cheap and abundantly available agro-residue, through process optimization and to monitor the effect of temperature shift operation on it. The potential of xylanase in saccharification of wheat straw was also investigated. The results showed that optimization of the fermentation process by one variable approach increased the enzyme yield from 402 to 4,986 IU/ml. Subsequently, optimization of nitrogen and carbon sources through response surface methodology led to high level xylanase production (7,295 IU/ml) which was 1.46-fold greater than one variable approach after 56 h of cultivation at 30 °C. Temperature shift operation during fermentation resulted in maximum xylanase production in lesser duration (48 h instead of 56 h). Enzymatic hydrolysis of the alkali pre-treated wheat straw with 500 IU xylanase alone released 173 ± 8 mg sugars/g whereas in combination with cellulase and β-glucosidase released 553 ± 12 mg sugars/g dry substrate in 6 h, indicating its potential in saccharification of the lignocellulosic substrate. Temperature shift operation is likely to be attractive for large scale industrial fermentation due to significant reduction in the operating cost. To our knowledge, this is the first report which showed the effect of temperature shift operation on xylanase production from bacteria. The xylanase production from *Bacillus* sp. in the present study is close to the highest titre reported in the literature. An enhanced xylanase production using wheat bran, a cheap and abundantly available agro-residue, will apparently reduce the enzyme cost, which would be beneficial for industry.

## Introduction

Xylan, the major renewable hemicellulosic polysaccharide of plant cell walls, forms an interphase between lignin and other polysaccharides. It is a heteropolymer consisting of β-1,4-linked xylopyranose backbone with side-linked groups namely arabinofuranosyl, acetyl and glucuronosyl residues. Its complete hydrolysis requires the cumulative action of endo-β-1,4-xylanase (EC 3.2.1.8), β-xylosidase (EC 3.2.1.37), and a series of enzymes that degrade side chain groups. Among these, the most important enzyme is endo-β-1, 4-xylanase, which cleaves the glycosidic bonds in the xylan backbone to produce xylo-oligosaccharides of various lengths and xylose (Polizeli et al. 2005). Xylanase has gained immense interest due to its biotechnological potential in xylitol and ethanol production, paper industry, production of xylo-oligosaccharides, food industry, textile industry, animal feed industry, etc. (Dhiman et al. 2008; Kuhad and Singh 1993; Sharma and Kumar 2013). Xylanases used in industry are produced mainly from bacteria and fungi (Ahmed et al. 2009).

The wide-scale industrial applications of xylanase require cost-effective production of the enzyme to make the process economical. This can partly be achieved by using cheaply available agro-industrial residues such as wheat bran (Azeri et al. 2010; Sa-Pereira et al. 2002). The optimization of culture medium is a pre-requisite for successful use of microorganisms in industrial biotechnology because its composition can significantly affect the product yield. The optimization by one factor at a time approach requires a considerable effort and time, besides difficulty in analyzing the interactive effects of various factors. However, statistical design techniques can aid in optimization of culture medium, as they reveal the interactions among the process parameters at varying levels. In addition, the optimal level of each parameter for a given target can be calculated. Response surface methodology (RSM) is a widely used statistical approach to improve the enzyme yield though the magnitude of increase may vary (Bocchini et al. 2002; Riswan Ali et al. 2013).

The steady-state fermentation may not result in maximum productivity. The unsteady-state fermentation by changing the operational parameters such as pressure, temperature, flow rate, etc. via externally forced methods may enhance enzyme yield from microorganisms (Yuan et al. 2005). Therefore, it would be pertinent to investigate the effect of temperature shift during submerged fermentation (SmF) on xylanase yield.

Lignocellulosic biomass can be utilized to produce ethanol, a promising alternative energy source for the limited crude oil (Kuhad and Singh 1993). Wheat straw, an abundant low value by-product of wheat production worldwide, is an attractive lignocellulosic material for ethanol production. Its main constituents are 35–45 % cellulose, 20–30 % hemicelluloses and 8–15 % lignin (Saha et al. 2005) and its composition may vary depending on the wheat species, soil, climate conditions, etc. The first step in the conversion of wheat straw into ethanol is its saccharification to produce reducing sugars. Enzymatic saccharification of wheat straw is more promising compared to chemical hydrolysis as it requires low energy and is environment friendly. However, there is limited accessibility of these enzymes to their substrates in the lignocellulose complex due to the presence of lignin. It has been shown that pre-treatment of lignocellulosic materials with chemicals (dilute acid, alkali, sodium borohydrate), steam explosion, biological, or a combination of these, can significantly enhance the accessibility of the enzymes to cellulose and hemicelluloses present in these materials (Çöpür et al. 2012; Govumoni et al. 2013; Gupta et al. 2011; Mosier et al. 2005). Pre-treatment of lignocellulosic biomass is, therefore, crucial before enzymatic saccharification. As xylan is the major hemicellulose in secondary plant cell walls, addition of xylanase during hydrolysis of wheat straw is expected to increase the yield of monomeric sugars.

The present study was envisaged to modulate the xylanase production from an alkaliphilic *B. pumilus* VLK-1 (isolated from soil in our lab) using cheap and abundantly available agro-residue wheat bran through process optimization and temperature shift operation so as to obtain a high yield of the enzyme at low cost for industrial applications. The potential of this xylanase in saccharification of alkali pre-treated wheat straw was also evaluated.

## Materials and methods

### Raw materials and chemicals

Lignocellulosic substrates (wheat bran and wheat straw) were obtained from the local market, dried and powdered. The chemicals used in this study were purchased from Sigma Chemicals (St. Louis, MO), Hi-Media Laboratories, Mumbai (India) and Merck Laboratories, India.

### Microorganism, maintenance and inoculum production

*Bacillus. pumilus* VLK-1 (GeneBank accession No. JQ350583), a potent xylanase producing alkaliphilic bacterial stain isolated in our laboratory from a soil sample collected from the vicinity of a plywood industry at Yamuna Nagar, Haryana, India. The bacterial strain was grown on xylan-agar medium containing (g/l): peptone 5.0, beef extract 3.0, xylan 1.0, and agar 20.0 (pH 7.0) followed by incubation at 37 °C for 24 h. The culture was stored at 4 °C and sub-cultured fortnightly. Inoculum was developed by inoculating the autoclaved nutrient broth in a conical flask with a loop full of the overnight grown culture of *B. pumilus* VLK-1 followed by incubation at 37 °C at 200 rpm.

### Assay of xylanase

The activity of xylanase was assayed according to the method of Bailey et al. (1992) by measuring the amount of reducing sugars (xylose equivalent) liberated from xylan using 3,5-dinitrosalicylic acid (Miller 1959). The reaction mixture containing 490 μl of 1 % birch wood xylan (Sigma) as substrate (prepared in 0.05 M Tris–HCl buffer, pH 8.0) and 10 μl of appropriately diluted enzyme extract was incubated at 60 °C for 10 min. The reaction was then terminated by adding 1.5 ml of 3,5-dinitrosalicylic acid reagent. A control was run simultaneously that contained all of the reagents but the reaction was terminated prior to the addition of enzyme. The reaction mixture was heated in a boiling water bath for 10 min followed by cooling in ice-cold water. The absorbance of the resulting color was measured against the control at 540 nm in a spectrophotometer. The amount of xylose produced was measured from its standard curve. One international unit (IU) of xylanase activity was defined as the amount of enzyme required to release 1 μmol of xylose per min under the specified assay conditions.

### Assay of cellulase

Cellulase activity [carboxymethyl cellulase (CMCase) and filter paper hydrolyzing activity (FPase)] was determined according to Ghosh (1987). One IU of cellulase activity was defined as the amount of enzyme required to release 1 μmol glucose per min under the specified assay conditions.

### Optimization of xylanase production by one variable at a time approach

Xylanase production in SmF was optimized in flasks (250 ml) containing 50 ml of basal medium comprising of 0.2 g/l MgSO_4_·7H_2_O, 1.0 g/l K_2_HPO_4_ and 2.0 % (w/v) wheat bran by varying one factor at a time. The enzyme production was carried out at 37 °C, pH 7.0, and 200 rpm with an inoculum size of 1.0 % (v/v). After 48 h of incubation, fermentation broth was centrifuged at 10,000×*g* for 15 min and the culture filtrate was collected for assay of xylanase activity as described above. The xylanase yield from *B. pumilus* VLK-1 under SmF conditions was optimized with respect to various parameters such as inoculum age (2–12-h-old culture), inoculum size (1.0–5.0 % v/v), cultivation time (0–72 h), cultivation temperature (30–45 °C), pH of the medium (4.0–11.0) and agitation rate (125–250 rpm) using one variable at a time approach. In addition to these factors, carbon and nitrogen source and additives were also optimized. Various carbon sources (1 % w/v) such as wheat bran, wheat straw, rice straw, birchwood xylan, oat spelt xylan, sugars (lactose, sucrose, xylose and glucose), carboxymethyl cellulose (CMC) and corn cob were tested as the sole substrate for xylanase production in the basal medium. The nitrogen source was optimized by supplementing the basal medium with different organic (yeast extract, tryptone, peptone, beef extract, and casein) and inorganic nitrogen (KNO_3_, NaNO_3_, Ca(NO_3_)_2_ and NH_4_Cl) sources at 0.5 % (w/v) each. The optimum concentrations of the best carbon and nitrogen sources were also determined. The effect of different additives (Tween 20, Tween 40, Tween 80, olive oil, glycerol, EDTA and Triton X-100) on xylanase production was investigated by individually supplementing these in the basal production medium at a final concentration of 0.1 % (w/v or v/v) with flask lacking any additive as the control. All the experiments were performed in triplicate and the average values have been reported.

### Optimization of xylanase production by statistical approach

The interaction among the four efficient factors, i.e. peptone (A), yeast extract (B), KNO_3_ (C) and wheat bran (D), selected from one variable approach, was investigated on xylanase production from *B. pumilus* VLK-1 through RSM using statistical software package Design Expert 8.0.1, Stat-Ease, Inc., USA. A 2^4^ full factorial central composite design (CCD) with 16 trials for factorial design, 8 trials for axial point, and 6 trials for replication of central point, leading to a set of 30 experiments was designed to optimize the concentrations of nitrogen source and carbon source for the production of xylanase from *B. pumilus* VLK-1. These 30 experiments were performed in triplicate and the average response value from each experiment was calculated.

### Effect of temperature shift on xylanase production

Temperature shift during fermentation is likely to affect xylanase production because the optimum temperatures for growth and enzyme production by the bacterial strain may be different. So, the effect of temperature shift during fermentation was investigated on xylanase production by *B. pumilus* VLK-1. The organism was cultivated in the optimized SmF medium in shake flasks which were then incubated in an orbital shaker. One set of flasks was incubated at 30 °C and another set at 37 °C for a period of 56 h. Third set of flasks was given temperature shift treatment by initially incubating it at 37 °C for a period of 4–12 h and then shifting the cultivation temperature to 30 °C up to a total period of 56 h. Samples were harvested from all the three sets of flasks at different time intervals starting from 24 h for monitoring xylanase production. The experiment was performed in triplicates and the average values have been given.

### Enzymatic saccharification of wheat straw

Wheat straw was washed, dried, milled and sieved to obtain a fine powder, which was used for enzymatic saccharification after alkali pre-treatment. The powdered wheat straw was treated with 0.1 N NaOH followed by incubation for 2 h at room temperature along with shaking. Thereafter, the wheat straw was thoroughly washed with tap water to neutral pH and filtered the contents through double-layered muslin cloth. The residue was dried overnight at 60 °C to a constant weight and used for saccharification. The alkali pre-treated wheat straw (1.0 g) was suspended at 2 % w/v consistency in 50 ml of 0.05 M sodium phosphate buffer pH 6.0, 1.0 ml of Tween 80, 500 IU xylanase (produced by *B. pumilus* VLK-1), 80 FPU of FPase and 160 U of β-glucosidase (Sigma) followed by incubation at 40 °C for 6 h in a rotary shaker at 100 rpm. An alkali untreated sample of wheat straw was also processed under identical conditions. A control was also run simultaneously in which no enzyme was added. After incubation, samples were centrifuged at 10,000×*g* for 15 min and the supernatants were analyzed for sugar content using 3,5-dinitrosalicylic acid. The efficiency of saccharification was evaluated by measuring the sugar yield (mg/g dry substrate). The saccharification was optimized with respect to different parameters.

## Results and discussion

In this study, extracellular xylanase was produced in basal medium under SmF from *B. pumilus* VLK-1, an alkaliphilic strain. The xylanase titre under unoptimized conditions was 402 IU/ml, which increased to 7,295 IU/ml (18.1-fold increase) through optimization of fermentation parameters by one variable approach and RSM. The potential of this enzyme in saccharification of wheat straw was studied.

### Optimization of xylanase production by one variable approach

Xylanase production was monitored using the basal medium (pH 7.0) inoculated with 1.0 % (v/v) of overnight grown secondary inoculum of *B. pumilus* VLK-1 and incubated at 37 °C under shaking at 200 rpm for 48 h by individually varying the factors viz. inoculum age and size, incubation time, cultivation temperature, initial pH of the production medium, carbon source, nitrogen source and additives.

### Age and size of inoculum

**Fig. 1:**
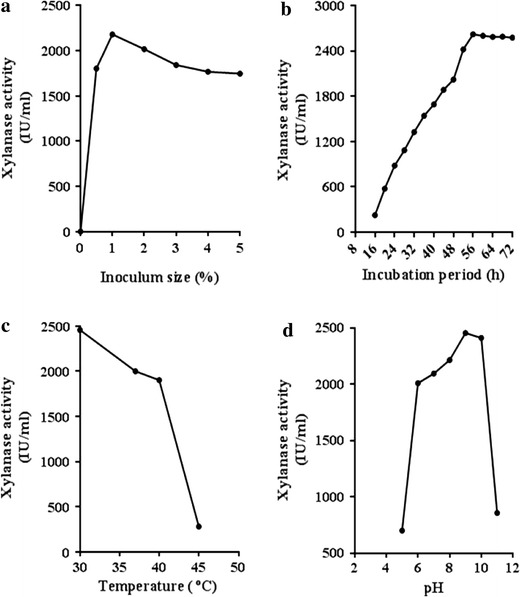
Effect of **a** inoculum size, **b** incubation period, **c** cultivation temperature, and **d** pH of the production medium on xylanase production by *Bacillus pumilus* VLK-1 in SmF. The enzyme was produced in basal medium and the above mentioned factors were varied one at a time

The effect of inoculum age was studied by measuring the xylanase titre after inoculating 50 ml of the production medium with 1 % (v/v) of 2–12-h-old secondary inoculum. The highest enzyme production was recorded during the log phase reaching maximum (3,056 IU/ml) at 8 h followed by a decline. The profile of xylanase activity as a function of inoculum size (1.0–5.0 % v/v) revealed maximum enzyme yield at 1.0 % (Fig. 1a). An optimal inoculum level is necessary for maintaining the balance between proliferating biomass and available nutrients to obtain maximum enzyme yield. A lower enzyme yield at higher inoculum level could result from faster nutrient consumption. Moreover, a high level of inoculum is not favorable to its application in industry. Most researchers have reported the use of 1.0–2.0 % (v/v) inoculum for hyper production of xylanase in SmF (Nagar et al. 2012; Sanghi et al. 2009).

### Cultivation period

Time course of xylanase production showed maximum enzyme activity after 56 h of incubation and thereafter, it remained more or less constant till 72 h (Fig. 1b). The optimum time resulting in maximum enzyme titre is likely to depend on several factors including the microbial strain. A survey of the literature revealed the highest enzyme production from *B. pumilus* SV-85 after 36 h (Nagar et al. 2010), *B. subtilis* ASH after 48 h (Sanghi et al. 2009), and *Bacillus* SSP-34 after 96 h (Subramaniyan and Prema 2000) of incubation. In the above reports, the activity of xylanase exhibited a decline after reaching a maximum value, which might be due to proteolysis of the enzyme. However, in the present study, though the incubation period for xylanase production from *B. pumilus* VLK-1 was longer than some other *Bacillus* sp. yet it did not decline after attaining the highest level. The rationale for this observation is not understood.

### Cultivation temperature

A record of xylanase activity at various cultivation temperatures showed the highest enzyme production at 30 °C (Fig. 1c). The optimum temperature for enzyme production by an organism may vary since it is likely to affect growth of the organism. The present findings are in accordance with those of Ratto et al. (1992) who observed highest xylanase production by *Bacillus circulans* at 30 °C using xylan. Other *Bacillus* sp. have been reported to exhibit maximum xylanase titre at 37 °C (Nagar et al. 2012), 50 °C (Subramaniyan and Prema 2000) and 55 °C (Annamalai et al. 2009). Anand et al. (2013) reported the production of an alkali-tolerant and thermostable xylanase from *Geobacillus thermodenitrificans* at 60 °C, however, the activity was only 2.75 U/ml.

### Cultivation pH

Each microorganism requires an optimum pH for its growth and activity. The initial pH of the medium may influence many enzymatic systems and the transport of enzymes across the cell membrane. Among a wide range of initial pH of the production medium tested, the optimum pH for xylanase production from *B. pumilus* VLK-1 was found to be 9.0 (Fig. 1d). A similar pH optimum was reported for xylanase production from *B. circulans* (Bocchini et al. 2002; Ratto et al. 1992), *Bacillus* sp. VI-4 (Yang et al. 1995) and *B. subtilis* (Annamalai et al. 2009). The optimum pH documented by other researchers was 6.0 (Nagar et al. 2010), 7.0 (Archana and Satyanarayana 1998; Sanghi et al. 2009) and more than 9.0 (Nagar et al. 2012). Thus, the optimum pH for enzyme production varies from one bacterial strain to another. In the present study, the bacterial strain is alkaliphilic capable of growing in the pH range of 6.0–10.0.

### Agitation rate

The highest xylanase production by *B. pumilus* VLK-1 was observed at an agitation rate of 200 rpm after 48 h of incubation. The enzyme yield was low if the agitation rate was increased or decreased beyond 200 rpm. This was consistent with earlier reports (Sanghi et al. 2009; Sepahy et al. 2011). The lower enzyme level under low agitation conditions may be attributed to the dissolved oxygen limitation for cell growth, improper mixing of media components and cell clumping.

### Carbon source

**Fig. 2:**
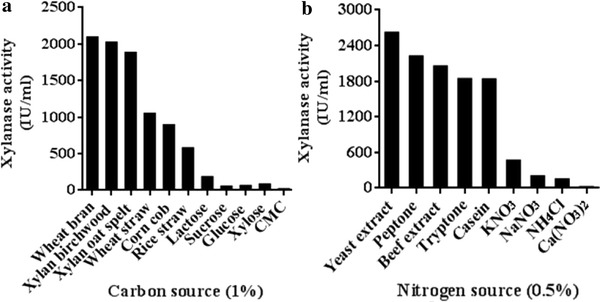
Effect of different **a** carbon sources and **b** nitrogen sources on xylanase production by *Bacillus pumilus* VLK-1 in SmF

Carbon source is an essential constituent of the fermentation medium, which affects cellular metabolism of the microorganism. The effect of various carbohydrates and agro-residues, each at a concentration of 1 % (w/v), was investigated on xylanase production by *B. pumilus* VLK-1. Among the different carbon sources tested, wheat bran supported the highest xylanase production followed by birchwood xylan, oat spelt xylan, wheat straw and rice straw (Fig. 2a). In control, the enzyme production was negligible. On varying the concentration of wheat bran from 1.0 to 8.0 % in the basal medium, the highest xylanase production was recorded at 4 %. The xylanase production was higher with wheat bran as carbon source as compared to pure xylan. It might be due to the fact that it contained 54 % carbohydrates (including xylan, pentoses and hexoses), 14 % protein, minerals, vitamins and amino acids (El-Sharnouby et al. 2012), which would support growth of the bacterium and hence, xylanase production. Wheat bran has been documented as the best carbon source for xylanase production from *Bacillus* species by several researchers (Archana and Satyanarayana 1998; Azeri et al. 2010; Irfan et al. 2012; Nagar et al. 2012; Sanghi et al. 2009). In contrast, higher production of a thermotolerant xylanase from *B. subtilis* in SmF was reported with cassava bagasse as compared to wheat bran as carbon source (Sugumaran et al. 2013). Anand et al. (2013) reported the production of an inducible xylanase with the same titre in the presence of xylan and wheat bran. Some researchers found birchwood or oat spelt xylan to support maximum enzyme titre (Annamalai et al. 2009; Giridhar and Chandra 2010; Sa-Pereira et al. 2002). However, it is uneconomical to use purified xylan as substrate for large scale production of xylanase. The use of wheat bran, a cheap and readily available agro-residue, is likely to reduce the cost of xylanase production, a desirable attribute for its application in industries.

### Nitrogen source

The influence of several organic and inorganic nitrogen sources (0.5 % each) was examined on xylanase production by *B. pumilus* VLK-1. Addition of organic nitrogen sources such as peptone, yeast extract, and beef extract in the basal media resulted in higher enzyme titre as compared to inorganic compounds (Fig. 2b). The highest xylanase production in yeast extract might be due to the fact that it is a rich source of vitamins and minerals required for growth of the bacterium. The measurement of enzyme production using different concentrations of peptone, yeast extract and KNO_3_ individually showed the highest enzyme titre at 0.2, 0.6 and 0.2 %, respectively. Further, the enzyme production was higher in the presence of a combination of 0.2 % peptone, 0.6 % yeast extract and 0.2 % KNO_3_ as compared to individual nitrogen sources. There is a lot of variability with regard to optimal nitrogen source for xylanase production by different bacterial strains. The highest xylanase titre from *Bacillus* sp. SSP-34 was recorded with 0.25 % each of yeast extract and peptone (Subramaniyan et al. 2001) whereas tryptone was the best nitrogen source for *B. circulans* AB16 (Dhillon and Khanna 2000). In some cases, maximum xylanase production from *Bacillus* sp. was obtained with yeast extract (Annamalai et al. 2009; Sugumaran et al. 2013) or a combination of yeast extract and tryptone (Giridhar and Chandra 2010; Sepahy et al. 2011).

### Effect of additives

The effect of various additives at a concentration of 0.1 % was examined on xylanase production. Addition of olive oil and Tween 80 individually to the basal medium stimulated the enzyme production by 37 and 38 %, respectively, whereas Tween 20 and Tween 40 did not show any significant effect as compared to the control (Table 1). Triton X-100 and EDTA were found to be inhibitory. These results are in line with earlier reports in which 0.1 % Tween 80 was found to enhance xylanase production (Giridhar and Chandra 2010). Nagar et al. (2010) observed stimulation of xylanase production from *B. pumilus* SV-85S by 0.2 % olive oil and 0.1 % Tween 80 but inhibition by a combination of these two additives.

**Table 1:** Effect of various additives on xylanase production by *B. pumilus* VLK-1

Additive	Xylanase activity (IU/ml)
Control	3,660 ± 21
Tween 20	3,661 ± 16
Tween 40	3,664 ± 11
Tween 80	3,980 ± 12
Olive oil	3,942 ± 10
Glycerol	2,939 ± 16
EDTA	2,927 ± 13
Triton X-100	567 ± 06

### Xylanase titre under optimized conditions through one variable approach

Xylanase production by *B. pumilus* VLK-1 increased from 402 to 4,986 IU/ml under the optimized conditions (1 % of 8-h-old secondary inoculum, incubation time 56 h, temperature 30 °C, pH 9.0, agitation rate 200 rpm, 4 % wheat bran as carbon source, a combination of 0.2 % peptone, 0.6 % yeast extract and 0.2 % KNO_3_ as nitrogen source, 0.1 % olive oil and 0.1 % Tween 80) through one variable approach.

### RSM for optimization of nitrogen and carbon source

The optimum combination of nitrogen source (peptone, yeast extract and KNO_3_) and carbon source (wheat bran) for xylanase production from *B. pumilus* VLK-1 in SmF was determined through RSM. Each variable was used at five coded levels (Table 2) and all the variables were coded as ‘0’ at the central point. The values of xylanase activity predicted by central composite design (CCD) and those observed experimentally are shown in Table 3. Responses of the CCD design were fitted with a polynomial quadratic equation. The overall polynomial equation for xylanase production was:

**Table 2:** Different coded level of variables of central composite design (CCD) for xylanase production by *B. pumilus* VLK-1 under submerged fermentation

Independent variables	Unit	Coded level of variables				
		−2	−1	0	+1	+2
Peptone	%	−0.17	0.05	0.28	0.5	0.73
Yeast extract	%	−0.25	0.1	0.45	0.08	1.15
KNO_3_	%	−0.17	0.05	0.28	0.5	1.0
Wheat bran	%	−1.0	1.0	3.0	5.0	7.0

**Table 3:** Full factorial design for xylanase production by *B. pumilus* VLK-1 under submerged fermentation

Run	Peptone	Yeast extract	KNO_3_	Wheat bran	Xylanase activity (IU/ml)	
					Observed	Predicted
1	0.725	0.45	0.275	3	2,881.2	3,144.96
2	0.275	0.45	0.275	3	6,667.3	6,667.3
3	0.05	0.8	0.05	1	1,262	1,601.93
4	0.05	0.1	0.05	1	780	799
5	0.05	0.8	0.5	1	3,884	3,990
6	0.275	0.45	−0.175	3	1,576	1,640.39
7	0.5	0.1	0.5	1	956	1,013.28
8	0.275	0.45	0.275	3	6,667.3	6,667.3
9	0.5	0.1	0.5	5	1,988	1,811.44
10	0.275	−0.25	0.275	3	2,560	2,462.06
11	0.5	0.1	0.05	5	4,489.2	4,362.77
12	−0.175	0.45	0.275	3	4,328	3,921.23
13	0.275	0.45	0.275	3	6,667.3	6,667.3
14	0.05	0.8	0.05	5	5,240	5,162.37
15	0.5	0.8	0.5	5	4,048.2	4,008.74
16	0.275	0.45	0.275	3	6,667.3	6,667.3
17	0.05	0.1	0.05	5	1,828.68	1,864.5
18	0.275	0.45	0.275	3	6,667.3	6,667.3
19	0.275	0.45	0.275	3	6,667.3	6,667.3
20	0.275	0.45	0.725	3	1,734.96	1,502.39
21	0.5	0.8	0.5	1	588	715.55
22	0.05	0.1	0.5	5	1,549.44	1,521.79
23	0.5	0.1	0.05	1	1,604	1,704.73
24	0.275	0.45	0.275	7	3,404.4	3,818
25	0.275	0.45	0.275	−1	16	−567.94
26	0.05	0.1	0.5	1	1,818	2,316.27
27	0.5	0.8	0.05	5	6,024	5,689.09
28	0.5	0.8	0.05	1	528.72	536
29	0.275	1.15	0.275	3	5,465.6	5,441.36
30	0.05	0.8	0.5	5	5,628	1,521.79

$$\begin{array}{c} Y=6,667.3-194.06\times A+750.03\times B-40.79\times C\\ +1,089.65\times D-492.89\times A\times B-552.15\times A\times C\\ +398.16\times A\times D+217.75\times B\times C+623.76\times B\times D\\ -464.97\times C\times D-783.55\times A^{2}-681.5\times B^{2}1,270.83\times C^{2}-1,257.15\times D^{2} \end{array}$$

where *Y* is xylanase activity, *A* is peptone, *B* is yeast extract, *C* is KNO_3_ and *D* is wheat bran.

The analysis of variance (ANOVA) for this quadratic model clearly showed that the linear terms of yeast extract and wheat bran; square terms of peptone, yeast extract, KNO_3_ and wheat bran, and all the interactive terms except of yeast extract and KNO_3_ had a significant effect on enzyme production (on the basis of their *p*-values where *p* ≤ 0.0001 is considered to be significant) (Table 4). The statistical significance of the model equation was evaluated by the F-test for analysis of variance (ANOVA) which showed that the regression was statistically significant with a 99 % confidence level. The model *F*-value of 117.58 (as shown by the Fisher’s test) implied that the model was significant. A low probability value for the regression model [(Prob > *F*) < 0.0001] confirmed that the model was statistically significant for the optimization of nitrogen source and wheat bran for xylanase production. The determination coefficient (*R*^2^) of the model was 0.99 indicating that the model could explain up to 99 % of the variability. Moreover, *R*^2^ value was in reasonable agreement with adjusted *R*^2^ (0.9825), which corrects the *R*^2^ value for sample size and number of terms in the model. The predicted *R*^2^ was 0.95. The Adequate precision measures the signal to noise ratio. A ratio >4 was desirable. The ratio of 33.72 indicated an adequate signal. So, the model was significant for the process. The response surface plots (3D and contour) were analyzed to determine interactions among the variables and the optimum value of each factor for maximum xylanase production. Each plot addressed the combined effect of two variables while the remaining variables were maintained at ‘0’ (central) level.

**Table 4:** Result of regression analysis for response surface quadratic model

Source	*F*-value	*p* value (Prob > *F*)	
Model	117.58	<0.0001	Significant
*A*: Peptone	9.89	0.0067	
*B*: Yeast extract	147.74	<0.0001	
*C*: KNO_3_	0.44	0.5186	
*D*: Wheat bran	311.83	<0.0001	
*AB*	42.53	<0.0001	
*AC*	53.38	<0.0001	
*AD*	27.76	<0.0001	
*BC*	8.30	0.0114	
*BD*	68.82	<0.0001	
*CD*	37.85	<0.0001	
*A* ^2^	184.27	<0.0001	
*B* ^2^	139.40	<0.0001	
*C* ^2^	484.74	<0.0001	
*D* ^2^	474.36	<0.0001	

**Fig. 3:**
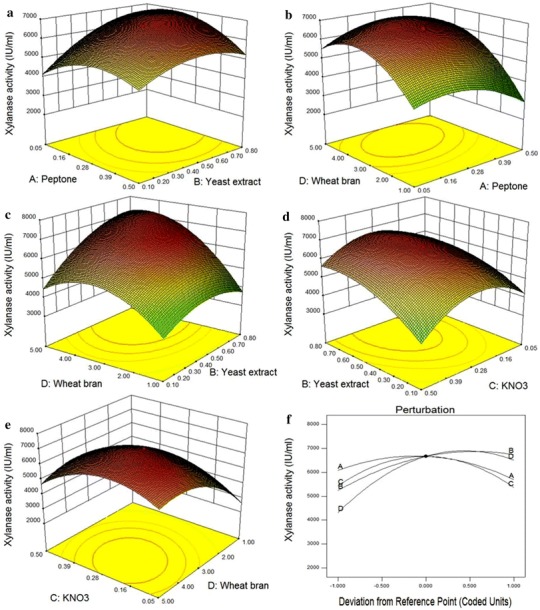
Response surface curves (3D/contour plots) showing interactions between **a** peptone and yeast extract; **b** wheat bran and yeast extract; **c** KNO_3_ and wheat bran; **d** peptone and wheat bran and **e** KNO_3_ and yeast extract and **f** is the perturbation plot showing simultaneous interaction of all the four factors on xylanase activity

Analysis of the 3D response surface plot between peptone and yeast extract (Fig. 3a) showed maximum xylanase activity at yeast extract concentration close to ‘+1’ level and peptone concentration between ‘−1’ and ‘0’ level. An increase in the concentration of yeast extract from ‘−1’ to ‘+1’ level led to an enhancement of xylanase activity whereas increase in the concentration of peptone above the central level led to a decline in the activity of xylanase.

The interaction between wheat bran and peptone is shown by a contour plot (Fig. 3b). It revealed maximum xylanase activity when peptone concentration was at its ‘0’ level and wheat bran concentration was between ‘0’ and ‘+1’ levels. The 3D plot between wheat bran and yeast extract (Fig. 3c) showed that both these factors at their ‘+1’ levels supported the highest xylanase activity and any decrease from this level led to decline in enzyme activity. Analysis of the contour plot between KNO_3_ and yeast extract (Fig. 3d) revealed maximum xylanase activity at the central level of KNO_3_ and for yeast extract between ‘0’ and ‘+1’ levels. The 3D response plot between wheat bran and KNO_3_ (Fig. 3e) was similar to KNO_3_ and yeast extract. The simultaneous effect of all the four variables on xylanase activity can be seen in perturbation graph (Fig. 3f).

The optimum values of the four factors suggested by the response surface model were 0.29 g peptone, 0.27 g KNO_3_, 0.77 g yeast extract and 4.53 g wheat bran for 100 ml of production medium. The concentrations of other medium components were kept same as in the original production medium. The model predicted 7,305.5 IU/ml of xylanase activity from the above optimized concentrations of the four factors. The predicted value was validated by determining xylanase production experimentally using the above optimized concentrations of the four factors and other parameters as in one variable approach. The experimental value of xylanase activity (7,295 IU/ml) was close to the predicted value. The xylanase production using RSM was 1.46-fold higher than one variable approach. A comparison of xylanase production from *B. pumilus* VLK-1 under SmF conditions revealed that it was higher than the enzyme titre from most of the microbial sources but close to the highest value reported from *Bacillus* sp. in the literature (Table 5). Riswan Ali et al. (2013) recorded xylanase production of 4.12 IU/ml from *Cellulomonas fimi* in SmF using tapioca stem in RSM optimized medium components.

**Table 5:** Comparison of xylanase production by different *Bacillus* sp.

Organism	Carbon source	Xylanase activity (IU/ml)	References
*Bacillus* sp. AG20	Wheat bran	3.5	Azeri et al. (2010)
*B. circulans*	Xylan	400	Ratto et al. (1992)
*B. circulans* D1	Xylan	1,901	Bocchini et al. (2002)
*B. licheniformis*	Wheat bran	756	Archana and Satyanarayana (1998)
*B. pumilus* B20	Wheat bran	313	Geetha and Gunasekaran (2010)
*B. pumilus* SV34S	Wheat bran	3,454	Mittal et al. (2012)
*B. pumilus* SV-85S	Wheat bran	2,995 ± 200	Nagar et al. (2012)
*B. pumilus* SV-205	Wheat bran	7,382 ± 1,200	Nagar et al. (2010)
*B. subtilis*	Oat spelt xylan	128	Annamalai et al. (2009)
*Bacillus* sp. V1-4	Birchwood xylan	49	Yang et al. (1995)
*B. subtilis*	Oat spelt xylan	18	Sa-Pereira et al. (2002)
*B. subtilis* ASH	Wheat bran	410	Sanghi et al. (2009)
*B. pumilus* VLK-1	Wheat bran	7,295	Present study

Thus, optimization experiments revealed maximum xylanase activity (7,295 IU/ml) after 56-h incubation at 30 °C with agitation of 200 rpm in the medium (pH 9.0) containing 4.53 % wheat bran, 0.29 % peptone, 0.77 % yeast extract, 0.27 % KNO_3_, 0.1 % olive oil, and 0.1 % Tween 80 and inoculated with 1 % of 8-h-old secondary inoculum.

### Modulation of xylanase production through temperature shift

The profile of xylanase production by *B. pumilus* VLK-1 under steady-state fermentation at 30 and 37 °C showed maximum enzyme activity after incubation for 56 h at both the temperatures; however, it was considerably higher at 30 °C than 37 °C (Table 6). This observation indicated that the optimum temperature for growth of the bacteria and enzyme production might be different. Hence, the effect of unsteady-state fermentation, which involved initial cultivation at 37 °C for different time periods (4,8 and 12 h) and subsequently shifting the temperature to 30 °C for the remaining time of fermentation up to a total duration of 56 h, was examined on xylanase production and the results are shown in Table 6. The data clearly showed that initial cultivation at 37 °C for 8 h and subsequently at 30 °C for the remaining period of fermentation led to nearly the same xylanase activity (7,106 IU/ml) after 48 h as that obtained (7,128 IU/ml) after 56 h under steady-state fermentation at 30 °C.

**Table 6:** Effect of cultivation temperature shift on xylanase production by *B. pumilus* VLK-1

Cultivation temperature	Cultivation period			
	24 h	36 h	48 h	56 h
30 °C	1,858 ± 35	3,261 ± 121	5,126 ± 152	7,128 ± 142
37 °C	1,370 ± 42	2,721 ± 78	4,692 ± 134	5,696 ± 112
4 h at 37 °C, then shifted to 30 °C	1,697 ± 58	2,845 ± 81	4,994 ± 187	6,238 ± 124
8 h at 37 °C, then shifted to 30 °C	1,898 ± 46	3,429 ± 72	7,106 ± 172	7,122 ± 108
12 h at 37 °C, then shifted to 30 °C	1,544 ± 28	2,735 ± 62	4,748 ± 156	6,018 ± 161

As a consequence of temperature shifting from 37 to 30 °C during cultivation, the time for maximum xylanase production was reduced from 56 to 48 h without any adverse effect on its activity. This approach of xylanase production might be beneficial for industry due to reduction in the operating cost. These results are similar to those of Yuan et al. (2005) who reported that unsteady-state fermentation by shifting the temperature of *Aspergillus niger* from 33 to 27 °C reduced the xylanase production time by 16 h without any adverse effect on its activity. However, we could not find any report in the literature on enhancement of bacterial xylanase production through temperature shift. This approach would likely be advantageous for large scale enzyme production due to reduction in the operating cost of the fermentor.

### Enzymatic saccharification of wheat straw

**Fig. 4:**
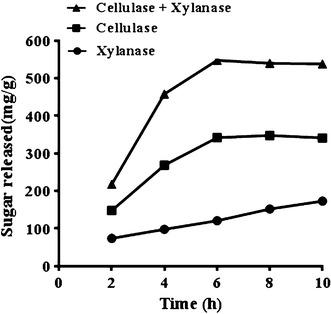
Enzymatic saccharification of alkali pre-treated wheat straw with xylanase alone (500 IU), mixture of FPase (80 FPU) and β-glucosidase (160 IU), and all the three enzymes (xylanase, FPase and β-glucosidase) per gram dry substrate for 6 h at 40 °C

Enzymatic saccharification of alkali pre-treated wheat straw (1.0 g) was carried out using 0.05 M phosphate buffer (pH 6.0) at 2 % (w/v) consistency with xylanase alone or its combination with FPase and β-glucosidase. This process was optimized by varying the different parameters one at a time. The optimum values of the various parameters were found as 0.05 M phosphate buffer (pH 6.0), 500 IU of xylanase, 80 FPU of FPase, 160 IU β-glucosidase, incubation temperature 40 °C, time 6 h, and 1 % Tween 80 on the basis of release of maximum amount of reducing sugars per gram dry substrate under these conditions. Treatment of alkali pre-treated wheat straw with xylanase alone, mixture of FPase and β-glucosidase, and all the three enzymes (xylanase, FPase and β-glucosidase) for 6 h at 40 °C increased the amount of reducing sugars as a function of time attaining a peak at 6 h releasing 173 ± 8, 396 ± 10 and 553 ± 12 mg sugars/g dry substrate, respectively (Fig. 4). It implied that addition of xylanase during hydrolysis of wheat straw enhanced the amount of sugars in the hydrolyzate for conversion into ethanol. This fact is consistent with earlier reports (Govumoni et al. 2013; Kumar et al. 2012; Saha et al. 2005; Tabka et al. 2006). The optimum time of hydrolysis in the present study, however, was only 6 h which might be due to higher doses of enzymes used compared to other reports.

The sugar release from lignocellulosic substrates apparently depends on several factors including pre-treatment conditions, concentrations of enzymes, temperature and time. The amount of sugars released in the present study were in accordance with Saha et al. (2005) who had reported the release of 565 ± 10 mg/g of acid pre-treated wheat straw on its saccharification with a mixture of cellulase, β-glucosidase, xylanase, and esterase at 45 °C for 72 h. The maximum release of glucose from acidic steam-pre-treated wheat straw was reported with a mixture of cellulase, xylanase and feruloyl esterase (Tabka et al. 2006).

On hydrolysis of alkali untreated wheat straw at 40 °C for 6 h, the amount of reducing sugars released in the presence of only xylanase, mixture of cellulase and β-glucosidase, and all the three enzymes (xylanase, FPase and β-glucosidase) was 39 ± 2, 74 ± 3, and 119 ± 6 mg/g, respectively (data not shown). These values were much lower as compared to the sugars released from alkali pre-treated wheat straw signifying the importance of pre-treatment in hydrolysis of lignocellulosic substrate. The main goal of pre-treatment is to increase the enzyme accessibility and hence to improve digestibility of cellulose (Mosier et al. 2005). Alkali pre-treatment was reported to improve the enzymatic saccharification of wheat straw by removing its lignin from the structure (Çöpür et al. 2012; Govumoni et al. 2013; Gupta et al. 2011). Lignin degradation in alkali treatment could be explained by breakage of ester linkages between lignin and xylan and deprotonation of lignin phenolic groups (Sun et al. 2005).

## Conclusion

In this study, the enhanced levels of xylanase (7,295 IU/ml) were produced from the alkaliphilic *B. pumilus* VLK-1 by the process optimization employing both one variable and statistical approach using cost-effective agro-residue, which was 18.1-fold higher than under unoptimized conditions. Application of temperature shift during fermentation shortened the time duration from 56 to 48 h for maximum xylanase titre resulting in cost-effective enzyme production. Thus, xylanase production could be profitably modulated through process optimization and temperature shift for use in industry. The xylanase produced in the present study was found to be effective in saccharification of alkali pre-treated wheat straw.
